# Muscle strength and incidence of depression and anxiety: findings from the UK Biobank prospective cohort study

**DOI:** 10.1002/jcsm.12963

**Published:** 2022-06-08

**Authors:** Verónica Cabanas‐Sánchez, Irene Esteban‐Cornejo, Solange Parra‐Soto, Fanny Petermann‐Rocha, Stuart R. Gray, Fernando Rodríguez‐Artalejo, Frederick K. Ho, Jill P. Pell, David Martínez‐Gómez, Carlos Celis‐Morales

**Affiliations:** ^1^ IMDEA Food Institute CEI UAM+CSIC Madrid Spain; ^2^ Institute of Cardiovascular and Medical Sciences University of Glasgow Glasgow UK; ^3^ PROFITH ‘PROmoting FITness and Health through physical activity’ Research Group, Sport and Health University Research Institute (iMUDS), Department of Physical Education and Sports, Faculty of Sport Sciences University of Granada Granada Spain; ^4^ Institute of Health and Wellbeing University of Glasgow Glasgow UK; ^5^ Facultad de Medicina Universidad Diego Portales Santiago Chile; ^6^ Department of Preventive Medicine and Public Health Universidad Autónoma de Madrid and CIBER of Epidemiology and Public Health (CIBERESP) Madrid Spain; ^7^ Cardiovascular and Nutritional Epidemiology Group IdiPAZ (La Paz University Hospital—Universidad Autónoma de Madrid) Madrid Spain; ^8^ Laboratorio de Rendimiento Humano, Grupo de Estudio en Educación, Actividad Física y Salud (GEEAFyS) Universidad Católica del Maule Talca Chile

**Keywords:** Grip strength, Muscular fitness, Anxiety, Depression, Mental disorders, Mental health

## Abstract

**Background:**

Depression and anxiety are the leading mental health problems worldwide; depression is ranked as the leading cause of global disability with anxiety disorders ranked sixth. Preventive strategies based on the identification of modifiable factors merit exploration. The aim of the present study was to investigate the associations of handgrip strength (HGS) with incident depression and anxiety and to explore how these associations differ by socio‐demographic, lifestyle, and health‐related factors.

**Methods:**

The analytic sample comprised 162 167 participants (55% women), aged 38–70 years, from the UK Biobank prospective cohort study. HGS was assessed at baseline using dynamometry. Depression and anxiety were extracted from primary care and hospital admission records. Cox proportional models were applied, with a 2 year landmark analysis, to investigate the associations between HGS and incident depression and anxiety.

**Results:**

Of the 162 167 participants included, 5462 (3.4%) developed depression and 6614 (4.1%) anxiety, over a median follow‐up period of 10.0 years (inter‐quartile range: 9.3–10.8) for depression and 9.9 (inter‐quartile range: 9.0–10.8) for anxiety. In the fully adjusted model, a 5 kg lower HGS was associated with a 7% (HR: 1.07 [95% CI: 1.05, 1.10]; *P* < 0.001) and 8% (HR: 1.08 [95% CI: 1.06, 1.10]; *P* < 0.001) higher risk of depression and anxiety, respectively. Compared with participants in the sex and age‐specific highest tertiles of HGS, those in the medium and lowest tertiles had an 11% (HR: 1.11 [95% CI: 1.04, 1.19]; *P* = 0.002) and 24% (HR: 1.24 [95% CI: 1.16, 1.33]; *P* < 0.001) higher risk of depression and 13% (HR: 1.13 [95% CI: 1.06, 1.20]; *P* < 0.001) and 27% (HR: 1.27 [95% CI: 1.19, 1.35]; *P* < 0.001) higher risk of anxiety, respectively. The association of HGS with depression was stronger among participants with average or brisk walking pace (vs. slow walking pace; *P*_interaction_ < 0.001). The association with anxiety was stronger in those participants aged ≥58 years (vs. ≤58 years; *P*_interaction_ = 0.002) and those living in more affluent areas (vs. deprived; *P*_interaction_ = 0.001).

**Conclusions:**

Handgrip strength was inversely associated with incident depression and anxiety. Because HGS is a simple, non‐invasive, and inexpensive measure, it could be easily used in clinical practice to stratify patients and identify those at elevated risk of mental health problems. However, future research should assess if resistance training aimed at increasing HGS can prevent the occurrence of mental health conditions.

## Introduction

Depression and anxiety are the leading mental health problems worldwide, affecting 322 and 264 million people, respectively.^1^ Depression is ranked, by the World Health Organization, as the leading cause of global disability (7.5% of all years lived with disability) with anxiety disorders ranked sixth (3.4%).^1^ A growing body of evidence indicates that people with depression or anxiety disorders are also at increased risk of several co‐morbidities,^2^ particularly cardiovascular diseases, and premature death^3^ and incur higher personal and healthcare costs.^4^ Therefore, given that traditional pharmacological treatments and psychotherapies offer limited effectiveness,^5^ preventive strategies based on the identification of modifiable factors merit exploration.

There is an increasing recognition of the importance of physical fitness as a potentially modifiable protective factor for the occurrence of mental health disorders.^6^ However, while low cardiorespiratory fitness has been elucidated as a risk factor for developing depression and anxiety,^7^ the role of muscular strength remains unclear.^8^, ^9^, ^10^ Handgrip strength (HGS) is a simple, non‐invasive, and low‐cost method that has been associated with several chronic diseases and all‐cause mortality across different age groups.^11^ Moreover, a recent study suggested that HGS can improve the prediction of existing cardiovascular risk scores.^11^, ^12^ Nevertheless, the temporal relationship between HGS and the development of anxiety disorders has been minimally investigated.^8^, ^9^, ^13^ Gordon *et al*.^9^ found that HGS was not significantly associated with incidence of generalized anxiety; however, the study population, which comprised 3234 older participants (≥50 years at baseline), limits the generalizability of the findings to the general population. Recently, Kandola *et al*.^13^ found a significant relationship between HGS and anxiety among the UK Biobank participants, but the incidence of anxiety was estimated using a self‐reported scale.

Moreover, despite the accumulating evidence for the prospective association between low muscular strength and depression, most of previous studies have focused on the recurrence of depressive symptoms in patients with history of depression rather than on depression onset in apparently healthy individuals. A recent narrative review of nine longitudinal studies reported that higher levels of muscular strength were associated with lower risk of developing depressive symptoms.^10^ However, this review highlighted the need for further studies on the topic, preferably spanning wider age ranges, and able to adjust for a wider range of potential confounders. To address gaps in the literature, we used data from UK Biobank, a large prospective cohort study, to investigate the associations of HGS with incident depression and anxiety and to explore whether these associations differ by socio‐demographic, lifestyle, and health‐related factors.

## Methods

### Participants

UK Biobank recruited more than 502 000 participants, aged 37–73 years, from the general population (5.5% response rate), between April 2007 and December 2010.^14^ Participants were invited to attend one of 22 assessment centres across England, Wales, and Scotland, where they completed a touchscreen questionnaire, and physical measurements and biological samples were also taken. Detailed procedures are described elsewhere.^15^ The study population for the present study comprised 162 167 UK Biobank participants (Supporting Information, *Figure* S1). The main outcomes in our analyses were depression and anxiety incidence. The exposure of interest was HGS, expressed in kg. A comprehensive list of potential confounders was considered including socio‐demographic, lifestyle, and health‐related factors.

The North West Multi‐centre Ethics Committee granted ethical approval to UK Biobank (reference: 16/NW/0274), and all participants provided written informed consent to participate in the study.

### Outcome measurement

To ensure comprehensive ascertainment, incident depression and anxiety were derived from linkage to both hospital inpatient and primary care records, which were available for ~45% of the UK Biobank cohort (*n* ≈ 23 000). An interim release of primary care records (up to 2016 or 2017 depending on the data supplier) was made available in 2019. This data set incorporates data from the GP system suppliers and contains coded clinical events, including diagnoses, procedures, consultations, and laboratory test. Hospital inpatient data were obtained via record linkage to Hospital Episode Statistics for England, Scottish Morbidity Record, and Patient Episode Database for Wales. This provides information on hospital admissions (up to 2018 or 2021 depending on the data supplier), including date of admission and discharge, and diagnosis, underlying conditions, and procedures during admission. Extensively detailed linkage procedures are available elsewhere (http://biobank.ndph.ox.ac.uk/showcase/showcase/docs/primary_care_data.pdf; https://biobank.ndph.ox.ac.uk/ukb/ukb/docs/HospitalEpisodeStatistics.pdf; and https://biobank.ndph.ox.ac.uk/ukb/exinfo.cgi?src=Data_providers_and_dates).

Thus, depression and anxiety were diagnosed by physicians, following the guidelines of the National Institute for Health and Care Excellence (https://pathways.nice.org.uk/pathways/common‐mental‐health‐disorders‐in‐primary‐care). We defined incident depression as the first record coded as the International Classification of Diseases, 10th Revision (ICD‐10), F32.0, F32.1, F32.2, F32.3, F32.8, F32.9, F33.0, F33.1, F33.2, F33.3, F33.4, F33.8, or F33.9. Incident anxiety was defined as the first record coded as ICD‐10 F40, F41, F42, or F43. As appropriate, follow‐up was censored at the most updated date of data extraction or at the date of the incident depression or anxiety, if this occurred earlier.

### Exposure measurement

Handgrip strength was assessed using a Jamar J00105 hydraulic hand dynamometer. This instrument measures grip isometric force (without movement); it can be adjusted to the hand size in five half‐inch increments, and the dual‐scale readout displays isometric grip strength from 0 to 90 kg. The dynamometer was calibrated before each measurement day, and the isometric grip strength was evaluated from a single 3 s maximum grip effort, separately for the right and left arms; the participant was sitting upright with their elbow by their side and flexed at 90° so that their forearm was facing forwards and resting on an armrest. The mean of both hands was derived and expressed in absolute units (kg). For the subsequent analyses, continuous HGS in kg, 5 kg decrease in HGS, and sex‐specific and age‐specific tertiles of HGS were used.

### Covariates

Socio‐demographic factors, including sex and ethnicity, were self‐reported at the baseline assessment visit. We calculated age from date of birth and date of baseline assessment. The Townsend Deprivation Index was derived from postcode of residence using census data on unemployment, non‐car ownership, non‐home ownership, and household overcrowding.^16^

Weight and height were measured by trained nurses, and body mass index (BMI; weight/height^2^) was used to classify participants as underweight (<18.5 kg/m^2^), normal weight (18.5–24.9 kg/m^2^), overweight (25.0–29.9 kg/m^2^), and obese (≥30 kg/m^2^). Physician‐diagnosed long‐term conditions were self‐reported and confirmed at nurse‐led interview at baseline. A number of morbidities, based on 43 commonly long‐term conditions,^17^ were categorized into none, one, and two or more.

Smoking status was self‐reported and categorized into never, former, and current smokers. Likewise, alcohol intake was recorded as (i) daily or almost daily, (ii) three or four times a week, (iii) once or twice a week, (iv) one to three times a month, (v) special occasions only, and (vi) never. Dietary intake information was self‐reported by a touchscreen questionnaire. Fruits and vegetables and red meat consumption were estimated as g/day and portion/day, respectively; processed meat and oily fish intake were collected using five categories: (i) never, (ii) less than once a week, (iii) 2–4 times a week, (iv) 5–6 times a week, and (v) once or more daily. Further details of these measurements can be found in the online protocol of UK Biobank (http://www.ukbiobank.ac.uk).

Participants indicated their usual walking pace as (i) slow, (ii) average/steady, or (iii) brisk. TV viewing was declared through the question: ‘In a typical day, how many hours do you spend watching TV?’ Sleep duration was self‐reported and categorized as short (<7 h/day), normal (7–9 h/day), and long (>9 h/day). Physical activity was obtained with the short‐form International Physical Activity Questionnaire, and total physical activity was calculated as the sum of walking plus moderate and vigorous activity, expressed as metabolic equivalents (MET‐min/week).^11^

### Statistical analyses

Baseline characteristics of the analytical sample by tertiles of HGS are presented as mean and standard deviation (SD) for continuous variables, and numbers of observations and percentages for categorical variables. We excluded from the analyses all individuals with any of the following conditions at baseline, based on retrospective record linkage: depression, anxiety, Parkinson's disease, schizophrenia, bipolar disorder, or substance abuse (*n* = 33 329); moreover, to reduce the potential influence of reverse causality, landmark analysis was performed excluding participants who developed depression or anxiety within the first 2 years of the follow‐up (*Figure* S1).

Cox proportional models were used to investigate the associations between HGS and risk of depression and anxiety, using follow‐up time as the timescale variable. The results were expressed as hazard ratios (HRs) and their 95% confidence intervals (CIs). To explore non‐linear associations, we used Cox proportional hazard models with exposure variables fitted on penalized cubic splines. Penalized spline is a variation of basis spline (B spline), with better model fit for most scenarios and comparable with the restricted cubic spline in other scenarios.^18^ We zeroed the estimated hazard ratio curves at the median of HGS. To check overall statistical significance, as well as non‐linearity of the exposures, we used likelihood ratio tests. Moreover, we also performed Cox regression to investigate the association of 5 kg decrements in HGS with incident depression and anxiety. In order to compare our results with previous studies, we also performed HRs for age‐specific and sex‐specific tertiles of HGS, using the highest tertile (Q3) as the reference category in all models. Additionally, we performed analyses using a sex‐specific cut‐off point for clinically relevant muscle weakness according to the Foundation for the National Institutes of Health Sarcopenia Project (HGS < 26 kg for men and <16 kg for women)^19^ and the European Working Group on Sarcopenia in Older People (EWGSOP; HGS < 27 kg for men and <16 for women).^20^

Three Cox models were fitted with incremental adjustment for potential confounders. Model 1 (minimally adjusted) was adjusted for socio‐demographic variables: age, sex, deprivation index, and ethnicity. Model 2 was additionally adjusted for lifestyle factors, including smoking status, alcohol intake, walking pace, TV viewing, sleep time, and dietary intake: fruits and vegetables, read meat, processed meat, and oily fish intake. Model 3 was further adjusted for health indicators: BMI and multimorbidity. We also conducted a sensitivity analysis by additionally adjusting for total physical activity. Further, sensitivity analyses were performed excluding participants with cardiovascular disease (CVD), cancer, or chronic obstructive pulmonary disease (COPD) at baseline.

To investigate whether the association between HGS (expressed per 5 kg decrement) and the study outcomes differ by socio‐demographic, lifestyle, and health‐related factors, the analyses were stratified by sex (male or female), age (below median or above median; <59 or ≥59 years), deprivation index (affluent or deprived), BMI (normal weight, overweight, or obese), number of morbidities (none, 1, or ≥2), smoking status (never, former, or current smoker), alcohol intake (not heavy or heavy intake), walking pace (brisk, average, or slow pace), TV viewing (>4 or ≤4 h/day), and sleep duration (normal, short, or long). Model 3 was applied to stratified analyses, excluding the grouping variable. Additionally, we fitted interaction terms between HGS and each of these factors using the fully adjusted model.

Analyses were performed with the statistical software Stata v.14 (StataCorp LP) and R statistical software v.3.6.2, using the packag*e survival*. The significance level was set at *P* < 0.05.

## Results

Of the 502 628 participants recruited to UK Biobank, 162 167 had full data available for the exposure, outcomes, and covariates, did not have prevalent mental health conditions at baseline, and were not excluded by the 2 year landmark analysis (*Figure* S1). The median follow‐up period was 10.0 years (inter‐quartile range: 9.3–10.8) for depression and 9.9 (inter‐quartile range: 9.0–10.8) for anxiety. Over the follow‐up period, 5462 (3.4%) participants developed depression and 6614 (4.1%) developed anxiety.

*Table* 1 shows the main characteristics of the participants by HGS tertile. Briefly, compared with participants in the highest tertile of HGS, those in the lowest tertile were more likely to be women, from deprived areas, and of non‐White ethnic background; they were older and had higher prevalence of obesity, multimorbidity, and slow walking pace. They also reported a lower intake of fruits and vegetables and red meat and spent more time watching TV. However, they were more likely to have lower alcohol consumption.

**Table 1:** Characteristics of the study sample by sex‐specific and age‐specific tertiles of handgrip strength

	All	Tertile of handgrip strength^a^		
		Highest	Medium	Lowest
*n*	162 167	52 426	52 431	57 310
Handgrip strength (kg), mean (SD)^b^	30.94 (11.04)	38.81 (10.44)	31.01 (8.53)	23.69 (8.32)
Socio‐demographic factors				
Sex^b^				
Female	89 567 (55.23)	28 390 (54.15)	29 903 (57.03)	31 274 (54.57)
Male	72 600 (44.77)	24 036 (45.85)	22 528 (42.97)	26 036 (45.43)
Age (years), mean (SD)^b^	56.64 (8.06)	55.96 (8.31)	56.62 (7.99)	57.28 (7.84)
Deprivation index^b^				
Lower (least deprived)	57 617 (35.53)	20 383 (38.88)	19 073 (36.38)	18 161 (31.69)
Middle	56 370 (34.76)	18 449 (35.19)	18 389 (35.07)	19 532 (34.08)
Higher (most deprived)	48 180 (29.71)	13 594 (25.93)	14 969 (28.55)	19 617 (34.23)
Ethnicity^b^				
White	155 267 (95.75)	51 058 (97.39)	50 689 (96.68)	53 520 (93.39)
Mixed background	1807 (1.11)	476 (0.91)	523 (1.00)	808 (1.41)
South Asian	3079 (1.90)	267 (0.51)	638 (1.22)	2174 (3.79)
Black	1556 (0.96)	548 (1.05)	451 (0.86)	557 (0.97)
Chinese	458 (0.28)	77 (0.15)	130 (0.25)	251 (0.44)
Lifestyle factors				
Smoking status^b^				
Never	97 888 (60.36)	31 082 (59.29)	31 750 (60.56)	35 056 (61.17)
Former	55 859 (34.45)	18 482 (35.25)	18 079 (34.48)	19 298 (33.67)
Current	8420 (5.19)	2862 (5.46)	2602 (4.96)	2956 (5.16)
Alcohol intake^b^				
Daily or almost daily	31 677 (19.53)	10 992 (20.97)	10 405 (19.85)	10 280 (17.94)
Three or four times a week	39 749 (24.51)	13 731 (26.19)	13 034 (24.86)	12 984 (22.66)
Once or twice a week	43 877 (27.06)	14 329 (27.33)	14 252 (27.18)	15 296 (26.69)
One to three times a month	18 176 (11.21)	5760 (10.99)	6001 (11.45)	6415 (11.19)
Special occasions only	17 082 (10.53)	4804 (9.16)	5301 (10.11)	6977 (12.17)
Never	11 606 (7.16)	2810 (5.36)	3438 (6.56)	5358 (9.35)
Walking pace^b^				
Slow pace	10 179 (6.28)	1846 (3.52)	2541 (4.85)	5792 (10.11)
Average pace	84 841 (52.32)	25 562 (48.76)	27 515 (52.48)	31 764 (55.42)
Brisk pace	67 147 (41.41)	25 018 (47.72)	22 375 (42.68)	19 754 (34.47)
TV viewing (h/day), mean (SD)^b^	2.75 (1.51)	2.63 (1.44)	2.73 (1.49)	2.88 (1.59)
Sleep time (h/day)^b^				
Short sleep (<7 h/day)	122 552 (75.57)	40 567 (77.38)	40 078 (76.44)	41 907 (73.12)
Normal (7–9 h/day)	37 573 (23.17)	11 339 (21.63)	11 772 (22.45)	14 462 (25.23)
Long sleep (>9 h/day)	2042 (1.26)	520 (0.99)	581 (1.11)	941 (1.64)
Fruits and vegetables intake (g/day), mean (SD)^b^	334.14 (189.85)	337.17 (185.32)	335.17 (187.98)	330.42 (195.5)
Processed meat intake^b^				
Never	15 188 (9.37)	4521 (8.62)	4960 (9.46)	5707 (9.96)
Less than once a week	51 317 (31.64)	17 029 (32.48)	16 906 (32.24)	17 382 (30.33)
Once a week	47 688 (29.41)	15 516 (29.60)	15 407 (29.39)	16 765 (29.25)
2–4 times a week	42 264 (26.06)	13 586 (25.91)	13 401 (25.56)	15 277 (26.66)
5–6 times a week	4586 (2.83)	1432 (2.73)	1405 (2.68)	1749 (3.05)
Once or more daily	1124 (0.69)	342 (0.65)	352 (0.67)	430 (0.75)
Red meat intake (portion/day), mean (SD)^b^	2.11 (1.42)	2.15 (1.41)	2.09 (1.38)	2.09 (1.45)
Oily fish intake^b^				
Never	17 030 (10.50)	4700 (8.97)	5304 (10.12)	7026 (12.26)
Less than once a week	53 302 (32.87)	17 309 (33.02)	17 254 (32.91)	18 739 (32.70)
Once a week	62 679 (38.65)	20 828 (39.73)	20 337 (38.79)	21 514 (37.54)
2–4 times a week	27 819 (17.15)	9157 (17.47)	9112 (17.38)	9550 (16.66)
5–6 times a week	1009 (0.62)	337 (0.64)	318 (0.61)	354 (0.62)
Once or more daily	328 (0.20)	95 (0.18)	106 (0.20)	127 (0.22)
Health‐related factors				
BMI categories^b^				
Underweight	715 (0.44)	152 (0.29)	229 (0.44)	334 (0.58)
Normal weight	53 595 (33.05)	16 503 (31.48)	18 335 (34.97)	18 757 (32.73)
Overweight	69 713 (42.99)	23 464 (44.76)	22 363 (42.65)	23 886 (41.68)
Obesity	38 144 (23.52)	12 307 (23.47)	11 504 (21.94)	14 333 (25.01)
Multimorbidity^b^				
None	62 957 (38.82)	22 301 (42.54)	20 933 (39.92)	19 723 (34.41)
One morbidity	54 592 (33.66)	17 894 (34.13)	17 848 (34.04)	18 850 (32.89)
Two or more morbidities	44 618 (27.51)	12 231 (23.33)	13 650 (26.03)	18 737 (32.69)

BMI, body mass index; SD, standard deviation.

Values are number (percentage), unless stated otherwise.

^a^ Sex‐specific and age‐specific tertiles of grip strength.

^b^ Significant differences (*P* < 0.05) between tertiles of grip strength estimated by *χ* ^2^ test and analysis of variance (ANOVA) for categorical and continuous variables, respectively.

**Figure 1:**
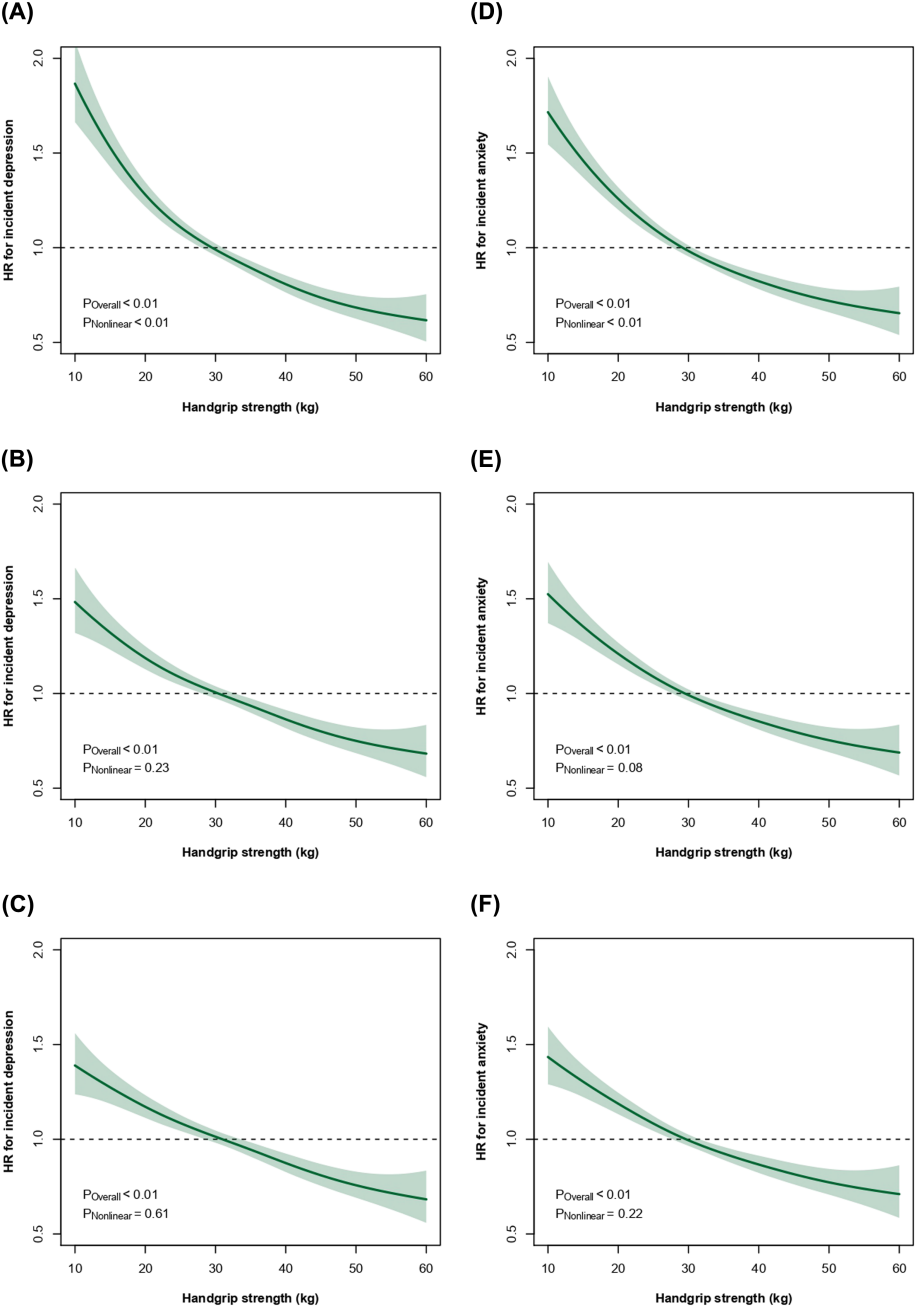
Penalized cubic spline analyses for the association of handgrip strength with (A–C) depression and (D–F) anxiety incidence. Data are presented as hazard ratio (HR) (thick blue line) and their 95% confidence interval (shaded areas). Participants with depression, anxiety, or mental health conditions at baseline were excluded from the analyses. A 2 year landmark analysis was applied. Analyses were adjusted for Model 1 (top plots) by socio‐demographic variables, including age, sex, deprivation index, and ethnicity; Model 2 (middle plots) was additionally adjusted by lifestyle factors, including smoking status, alcohol intake, walking pace, TV viewing, sleep time, and dietary intake (fruits and vegetables, read meat, processed meat, and oily fish intake); and Model 3 (bottom plots) was additionally adjusted by health markers, including body mass index and multimorbidity.

*Figure* 1 shows the association of HGS with incident depression. There was no strong evidence of a non‐linear association between HGS and incident depression. Although the magnitude of the association was slightly attenuated when the models were incrementally adjusted for lifestyle and health‐related factors, the association remained linear (*Figure* 1). The risk of incident depression was 13% higher per 5 kg decrement in HGS (HR: 1.13 [95% CI: 1.10, 1.15]). The associations were slightly attenuated when analyses were adjusted for lifestyle (HR: 1.08 [95% CI: 1.06, 1.10]) and health‐related (HR: 1.07 [95% CI: 1.05, 1.10]) factors (*Table* 2). When the risk of depression was estimated based on tertiles of HGS, those in the middle and lowest tertiles had a 14% and 39% higher risk of depression compared with those in the highest tertile. When the analyses were adjusted for lifestyle and health‐related factors, the associations were attenuated but remained significant (*Table* 2).

**Table 2:** Risk of depression and anxiety incidence according to HGS

Handgrip strength	Depression incidence			
	No. of participants/incidence	Model 1	Model 2	Model 3
		HR (95% CI)	HR (95% CI)	HR (95% CI)
Tertile 3 (highest)	52 426/1508	Ref.	Ref.	Ref.
Tertile 2 (medium)	52 431/1715	**1.14 (1.06, 1.22)**	**1.11 (1.04, 1.19)**	**1.11 (1.04, 1.19)**
Tertile 1 (lowest)	52 310/2239	**1.39 (1.29, 1.48)**	**1.26 (1.18, 1.35)**	**1.24 (1.16, 1.33)**
HR for tertile trend	162 167/5462	**1.18 (1.14, 1.22)**	**1.13 (1.09, 1.16)**	**1.11 (1.08, 1.15)**
*P* for trend		**<0.001**	**<0.001**	**<0.001**
HR per 5 kg lower HGS	162 167/5462	**1.13 (1.10, 1.15)**	**1.08 (1.06, 1.10)**	**1.07 (1.05, 1.10)**
*P* value		**<0.001**	**<0.001**	**<0.001**

	Anxiety incidence			
	No. of participants/incidence	Model 1	Model 2	Model 3
		HR (95% CI)	HR (95% CI)	HR (95% CI)
Tertile 3 (highest)	52 426/1831	Ref.	Ref.	Ref.
Tertile 2 (medium)	52 431/2117	**1.15 (1.08, 1.23)**	**1.13 (1.06, 1.21)**	**1.13 (1.06, 1.20)**
Tertile 1 (lowest)	57 310/2666	**1.37 (1.29, 1.45)**	**1.30 (1.22, 1.38)**	**1.27 (1.19, 1.35)**
HR for tertile trend	162 167/6614	**1.17 (1.14, 1.21)**	**1.14 (1.11, 1.17)**	**1.13 (1.09, 1.16)**
*P* for trend		**<0.001**	**<0.001**	**<0.001**
Per 5 kg lower HGS	162 167/6614	**1.12 (1.10, 1.14)**	**1.10 (1.07, 1.12)**	**1.08 (1.06, 1.10)**
*P* value		**<0.001**	**<0.001**	**<0.001**

CI, confidence interval; HGS, handgrip strength; HR, hazard ratio.

Model 1 was adjusted for age, sex, deprivation index, and ethnicity. Model 2 was adjusted for Model 1 plus lifestyle factors, including smoking status, alcohol intake, walking pace, TV viewing, sleep time, and dietary intake (fruits and vegetables, red meat, processed meat, and oily fish intake). Model 3 (fully adjusted) was adjusted as in Model 2 plus body mass index and multimorbidity. Significant values (*P* < 0.05) are in bold.

The associations between HGS and incident anxiety are presented in *Table* 2 and *Figure* 1. Again, there was no evidence of a non‐linear association between HGS and incident anxiety (*Figure* 1). The risk for anxiety was 12% higher per 5 kg decrement in HGS (HR: 1.12 [95% CI: 1.10, 1.14]), and compared with the highest tertile for HGS, those in the middle and lowest tertiles of HGS had a 15% (HR: 1.15 [95% CI: 1.08, 1.23]) and 37% (HR: 1.37 [95% CI: 1.29, 1.45]) higher anxiety incidence (*Table* 2). The associations were attenuated, but remained significant, when the analyses were adjusted for lifestyle and health‐related factors (*Table* 2).

When the analyses were further adjusted for total physical activity, the associations between HGS and incident depression and anxiety were very similar (*Figure* S2 and *Table* S1). Sensitivity analyses excluding people with CVD, cancer, or COPD at baseline yielded almost identical results on the association of HGS with depression and anxiety incidence (*Figure* S3 and *Table* S2).

Muscle weakness, defined using previously derived cut‐off points,^11^, ^19^ was associated with an increased risk of incident depression (HR: 1.16 [95% CI: 1.06, 1.27]) and anxiety (HR: 1.24 [95% CI: 1.14, 1.35]), in the fully adjusted models (Model 3; *Table* S3). Virtually identical results were observed when EWGSOP classification was used to define muscle weakness (*Table* S3).

**Figure 2:**
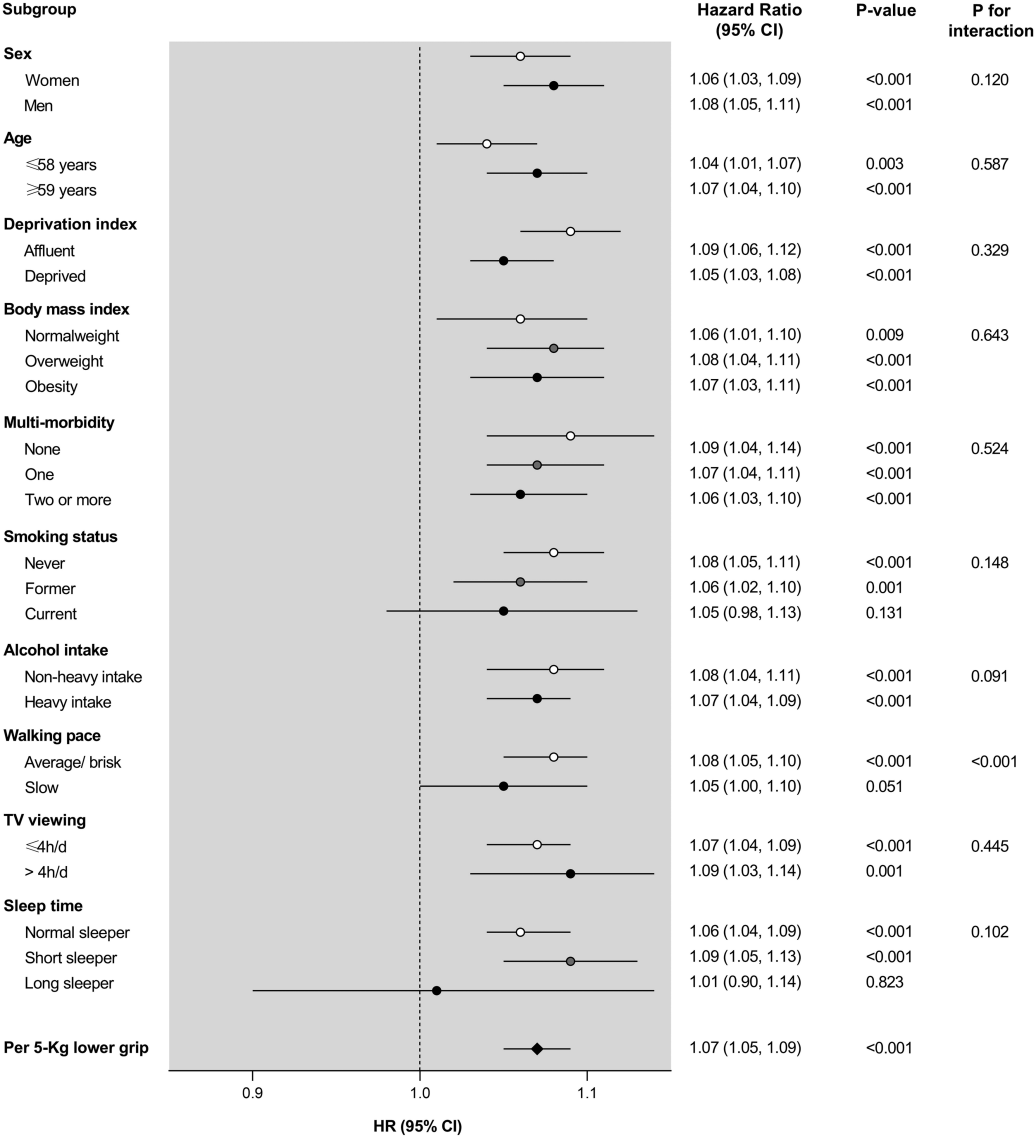
Depression incidence per 5 kg lower handgrip strength stratified by socio‐demographic, lifestyle, and health‐related factors. Analyses were adjusted for age, sex, deprivation index, ethnicity, smoking status, alcohol intake, walking pace, TV viewing, sleep time, dietary intake (fruits and vegetables, red meat, processed meat, and oily fish intake), body mass index, and multimorbidity, eliminating as co‐variable the grouping variable in each case. CI, confidence interval; HR, hazard ratio.

**Figure 3:**
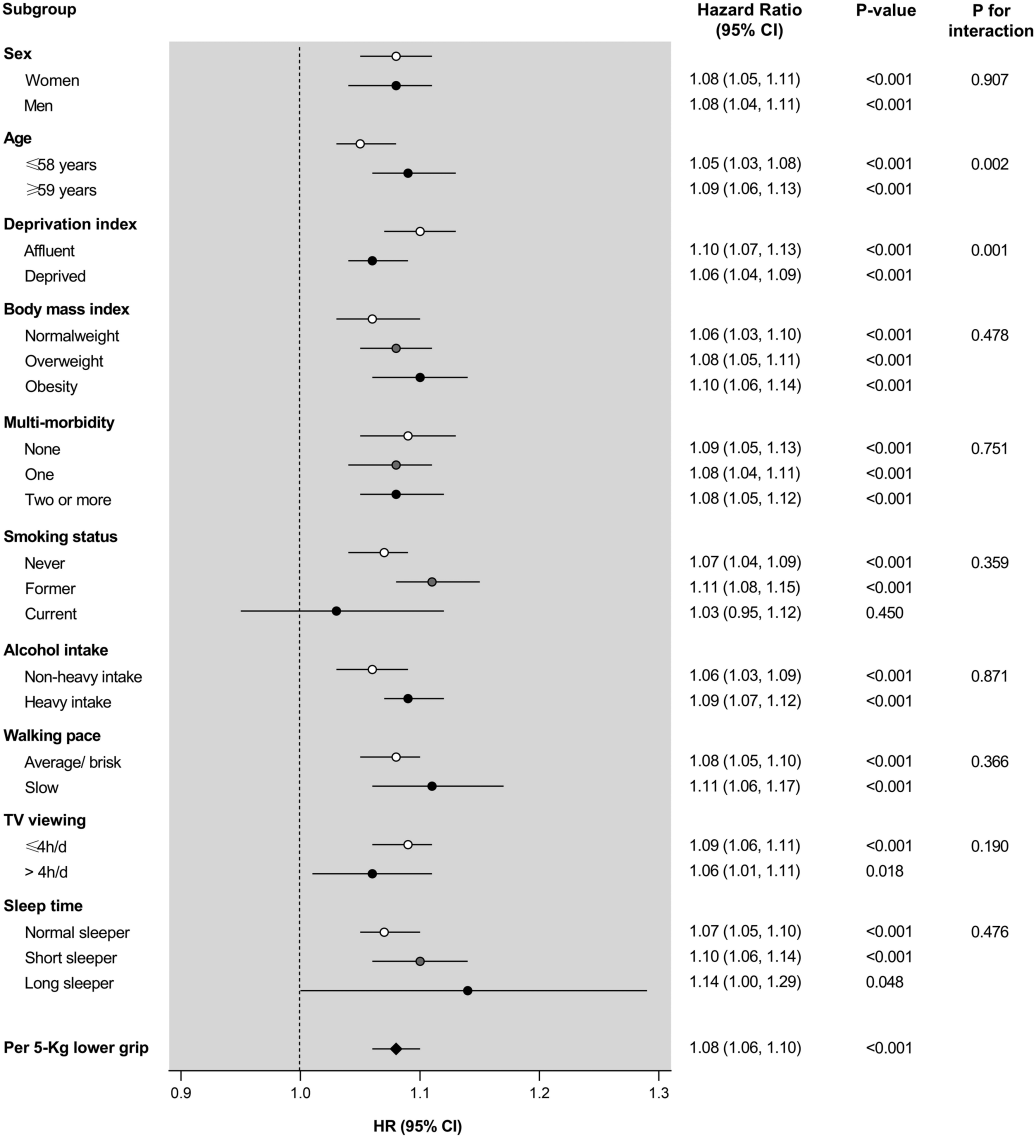
Anxiety incidence per 5 kg lower handgrip strength stratified by socio‐demographic, lifestyle, and health‐related factors. Analyses were adjusted for age, sex, deprivation index, ethnicity, smoking status, alcohol intake, walking pace, TV viewing, sleep time, dietary intake (fruits and vegetables, red meat, processed meat, and oily fish intake), body mass index, and multimorbidity, eliminating as co‐variable the grouping variable in each case. CI, confidence interval; HR, hazard ratio.

When the analyses on depression were stratified by socio‐demographic, lifestyle, and health‐related factors, no significant interactions were observed, with the exception of walking pace (*P*_interaction_ < 0.001), such that the risk of incident depression per 5 kg decrement in HGS was slightly higher among those with average or brisk walking pace than those with slow walking pace (*Figure* 2). For incident anxiety, the association with HGS was stronger among older participants (*P*_interaction_ = 0.002) and those living in more affluent areas (*P*_interaction_ = 0.001) (*Figure* 3).

## Discussion

The main finding of the present study is that lower HGS was associated with a higher risk of incident depression and anxiety in a large cohort of adults free of major mental health disorders. Potential socio‐demographic, health‐related, and lifestyle confounders explained relatively little of these associations. These findings may have important public health implications, as maintaining muscular strength may be protective against developing depression and anxiety. However, causality cannot be tested in the current study, and so there is a need for randomized controlled trials to investigate the effect of muscle strengthening exercise training on anxiety and depression.

Although a cross‐sectional association between muscle strength and depressive symptoms has been previously reported,^21^ there is limited and conflicting evidence from prospective studies.^10^ Bertoni *et al*.^22^ found that neither baseline nor incident muscle weakness (estimated by HGS) was associated with the onset of depressive symptoms among 6526 adults, aged ≥70 years, from 12 European countries. Conversely, a lower HGS was associated with the development of depressive symptoms among older adults in cohorts from England (3862 adults aged 64.4 ± 8.3 years)^23^ and Italy (970 elderly subjects ≥65 years).^24^ Similarly, McDowell *et al*.^25^ revealed that the middle and highest HGS tertiles had 32.1% and 32.8% lower odds of reporting incident depression over 2 year follow‐up, among a sample of 4104 adult (≥50 years) residents in Ireland. Likewise, lower HGS was associated with depressive symptoms after 1 year (adjusted odds ratio: 1.13 [1.01–1.27] per 1‐SD decrease) in a prospective cohort of 4314 Japanese individuals aged 40–79 years.^26^ Finally, a prospective cohort study, which enrolled 8470 Chinese adults aged ≥45 years who were followed up for 3.7 years, reported a 13% lower risk for severe depressive symptoms per 1‐SD increase in absolute HGS.^27^ However, these studies were largely conducted on small study populations predominantly composed of older adults, and depression was ascertained from self‐report rather than clinical diagnoses. A recent study using UK Biobank participants reported that low and medium HGS was associated with 1.41 and 1.12 higher odds of depression incidence, estimated by questionnaire (Patient Health Questionnaire‐9).^13^ Therefore, our study adds to the existing evidence by demonstrating an inverse association between HGS and subsequent onset of physician‐diagnosed depression in a large cohort of both middle aged and older adults.

In relation to anxiety, there is existing evidence of an association between low HGS and persistence of anxiety disorders,^28^ but the relationship between HGS and subsequent onset of anxiety remains largely unexplored. Carvalho *et al*.^8^ found no evidence that muscle weakness (HGS < 30 kg for men and <20 kg for women) was associated with incident anxiety among 5271 participants of the Irish Longitudinal Study on Ageing. Based on 3234 participants from the same cohort, Gordon *et al*.^9^ showed that 1‐SD higher HGS was non‐significantly associated with 24.2% lower odds of incident generalized anxiety disorders. In contrast, we found that, compared with the highest tertiles of HGS, participants in the medium and lowest tertiles had 13% and 27% higher risk of incident anxiety, respectively. Moreover, supplementary analyses revealed muscle weakness (HGS < 26 kg for men and <16 kg for women) to be associated with a 24% higher risk of incident anxiety. Similarly, Kandola *et al*.^13^ found that low and medium HGS was associated with 1.38 and 1.14 higher odds of anxiety incidence, self‐reported through the Generalized Anxiety Disorder‐7 scale. Our results add evidence to the study by Kandola *et al*.,^13^ because we used quality information derived from both hospital and primary care records, rather than self‐reported information. However, given the limited number of studies to date, further research on the potential protective effect of HGS on anxiety onset is needed.

Interestingly, we found slight moderation effects by some socio‐demographic, lifestyle, and health‐related factors on the associations between HGS and incidence of depression and anxiety. Regarding depression onset, we found a significant interaction term between HGS and walking pace, so that the relationship between HGS and incident depression was stronger among those with average or brisk walking pace, in comparison with those with slow walking pace. It has been reported that individuals with slow gait speed are at higher risk of developing depressive symptoms; mobility impairments limit the ability to perform activities of daily living, undermine self‐esteem, and decrease social interaction, which may result in the onset of depression.^29^ Therefore, it is possible that the participants in our study with low slow walking pace were already at increased baseline risk of depression, reducing the potential additional effect of low HGS. On the other hand, the association between HGS and incident anxiety was stronger among participants aged ≥59 years. This concurs with previously reported findings showing that the effect of HGS on mental health is greater among older participants.^21^, ^30^ Similarly, a previous meta‐analysis demonstrated that the effectiveness of strength training in the treatment of mood disorders is greater among patients older than 60 years.^31^ A possible reason may be that HGS strongly correlates with functional capabilities and cognitive function in the elderly.^32^ Moreover, we found stronger associations between HGS and incident anxiety among participants living in more affluent areas. A recent review by Linder *et al*.^33^ demonstrated that the negative consequences of depression and anxiety are greater among individuals with lower socio‐economic status. Therefore, the beneficial effect of HGS on incident anxiety may not be as great among disadvantaged communities, where access to treatment or prevention programmes is more limited.^33^

The mechanisms underpinning the associations of HGS with the development of depression or anxiety are largely unclear, but some hypotheses involving neurophysiological and psychosocial pathways could be speculated. Brain functional and structural alterations have been previously informed in patients with anxiety and depression; the most consistently affected area in people with mood disorders is the hippocampus, an area implicated in emotional processing and stress regulation.^34^, ^35^ Substantial evidence also demonstrated that people with depression or anxiety have decreased levels of BDNF, a marker of neuronal growth and plasticity.^36^ Meanwhile, greater HGS has been positively associated with hippocampal volume, white matter integrity, and neurocognitive functioning,^37^ and resistance training is known to increase BDNF levels,^38^ which might prevent anxiety and depressive symptoms. Moreover, there are several lines of evidence suggesting that chronic low‐grade inflammation may play an important role in the pathophysiology of mood disorders, because elevated levels of pro‐inflammatory markers [i.e., interleukin (IL)‐6, IL‐1, tumour necrosis factor‐α, or C‐reactive protein] have been described among patients with mood disorders.^39^ In response to muscle contractions, skeletal muscle tissues release several cytokines and myokines into the circulation, which anti‐inflammatory properties could protect against the risk of mood disorders.^39^ Besides, chronic oxidative stress was associated with an increased risk for mental conditions, but a recent meta‐analysis found resistance exercise associated with reductions on indicators of oxidative stress and increases in antioxidant levels.^40^ From a psychosocial perspective, engaging in health behaviours, such as exercise, increases feelings of self‐efficacy, self‐esteem, and social support, which may contribute to prevent mental health disorders; specifically, higher HGS has been linked to increased quality of life, mobility, and engagement in daily activities.^34^ Thus, it is possible that people with higher HGS are more likely to engage in more social activities and receive more social support, which, in turn, may lead to lower risk of developing depression or anxiety.

Overall, we found consistent positive associations of HGS with lower risk of incident depression and anxiety, which highlights the importance of maintaining healthy levels of strength across the lifespan. Therefore, the promotion of resistance exercise training, which reduces functional limitations and exerts anxiolytic effects,^41^ could be an effective strategy not only to reduce symptom severity among people with established mental health problems but also to protect the general population from developing them. Previous evidence from randomized controlled trials supports the effectiveness of resistance training programmes to reduce anxiety^41^ and depressive symptoms^42^ in people with mental health disorders. However, further work is needed to determine if resistance exercise can reduce incidence of depression and anxiety in general population.

### Strengths and limitations

Strengths of the current study include its prospective design, large sample, and the detailed assessment of the population, which allowed for conservative adjustment as well as subgroup analyses. Moreover, the main outcomes used in this study—depression and anxiety—were ascertained using linkage to primary care records, instead of self‐reported outcomes, and the exposure—HGS—was objectively measured by trained staff using validated methods and standard operating procedures. In addition, a wide range of potential confounders, covering socio‐demographic, lifestyle, and health‐related factors, could be controlled for in the analyses. However, this study is not without limitations. UK Biobank is relatively representative of the general UK population; therefore, summary statistics should not be generalized.^43^ Further, most of socio‐demographic, lifestyle, and health‐related factors were self‐reported, which could obscure the risk estimate due to recall bias. Moreover, although primary care and hospital records (based on diagnosis by physicians) may be a higher quality source of information than self‐reported measures, the diagnosis of depression and anxiety may be under‐reported so that the incidence of non‐severe symptoms of depression or anxiety may not have been recorded. Finally, although we excluded participants with relevant mental morbidities at baseline and undertook 2 year landmark analysis, residual confounding is possible in any observational study, reverse causation is still possible, and causality cannot be inferred from association.

### Conclusions

In conclusion, this study found an inverse association between HGS and incident depression and anxiety in a large prospective cohort of adults free of mental health problems at baseline. Socio‐demographic, health‐related, and lifestyle factors demonstrated slight moderation effects on the study relationships. These findings may have important clinical and public health implications. First, given that HGS is a simple, non‐invasive, and inexpensive measure, it could be easily used in clinical practice to stratify patients and identify those at elevated risk of mental health problems. Secondly, resistance training should be investigated in randomized controlled trials to determine its effects on incident anxiety and depression.

## Funding

UK Biobank was established by the Wellcome Trust, Medical Research Council, Department of Health, Scottish government, and Northwest Regional Development Agency. It also had funding from the Welsh Assembly Government and the British Heart Foundation. This research has been conducted using the UK Biobank Resource (Application No. 7155). V.C.‐S. is supported by the Spanish Ministry of Science, Innovation and Universities (Ministerio de Ciencia, Innovación y Universidades; IJC2018‐038008‐I). I.E.‐C. is supported by the Spanish Ministry of Economy and Competitiveness (Ministerio de Economía y Competitividad; RTI2018‐095284‐J‐100). D.M.‐G. is supported by a ‘Ramon y Cajal’ contract (RYC‐2016‐20546).

## Ethics statement

This study was performed under generic ethical approval obtained by UK Biobank from the National Health Service National Research Ethics Service (Approval Letter Ref. 11/NW/0382, 17 June 2011). Participants were not involved in the design, conduct, reporting, or dissemination plans of the research. The authors of this manuscript certify that they comply with the ethical guidelines for authorship and publishing in the *Journal of Cachexia, Sarcopenia and Muscle*.

## Author contributions

V.C.‐S., I.E.‐C., D.M.‐G., and C.C.‐M. contributed to the conception and design of the study. V.C.‐S., F.K.H., and C.C.‐M. conducted the statistical analysis. V.C.‐S., I.E.‐C., D.M.‐G., and C.C.‐M. wrote the first draft. All authors critically revised the manuscript. V.C.‐S., J.P.P., D.M.‐G., and C.C.‐M. are the guarantors of the manuscript and accept full responsibility for the work and/or the conduct of the study, had access to the data, and controlled the decision to publish.

## Conflict of interest

All the authors have completed the ICMJE uniform disclosure form at www.icmje.org/coi_disclosure.pdf and declare the following: UK Biobank was established by the Wellcome Trust medical charity, Medical Research Council, Department of Health, Scottish government, and Northwest Regional Development Agency; no financial relationships with any organizations that might have an interest in the submitted work in the previous 3 years; and no other relationships or activities that could appear to have influenced the submitted work.

## Supporting information


**Figure S1.** Flow chart for the selection of the final sample
**Figure S2.** Penalized cubic splines analyses for the association of HGS with (A) depression, and (B) anxiety incidence, additionally adjusted for physical activity. Data is presented as hazard ratio (thick blue line) and their 95% CI (shaded areas). Participants with depression, anxiety or mental health conditions at baseline were excluded from the analyses. A 2‐years landmark analysis was applied. Analyses were adjusted for age, sex, deprivation index, ethnicity, smoking status, alcohol intake, walking pace, TV viewing, sleep time, dietary intake (fruit and vegetables, red meat, processed meat and oily fish intake), body mass index, multimorbidity, and total physical activity (METs/min/week).).
**Table S1**. Risk of depression and anxiety incidence according to HGS, additionally adjusted for total physical activity
**Figure S3.** Penalized cubic splines analyses for the association of HGS with (A) depression, and (B) anxiety incidence, excluding from analyses participants with cardiovascular disease (CVD), cancer, and chronic obstructive pulmonary disease (COPD). Data is presented as hazard ratio (thick blue line) and their 95% CI (shaded areas). Participants with depression, anxiety or mental health conditions at baseline were excluded from the analyses. A 2‐years landmark analysis was applied. Analyses were adjusted for age, sex, deprivation index, ethnicity, smoking status, alcohol intake, walking pace, TV viewing, sleep time, dietary intake (fruit and vegetables, red meat, processed meat and oily fish intake), body mass index, and multimorbidity.
**Table S2**. Risk of depression and anxiety incidence according to HGS, after excluding participants with cardiovascular disease (CVD), cancer, and chronic obstructive pulmonary disease (COPD)
**Table S3**. Risk of depression and anxiety incidence according to muscle weakness and sarcopenia
