# Supplementary material for: Muscle strength and incidence of depression and anxiety: findings from the UK Biobank prospective cohort study
Source: J Cachexia Sarcopenia Muscle. 2022 Jun 8;13(4):1983–94. doi: 10.1002/jcsm.12963 (PMC9398224; doi:10.1002/jcsm.12963)
Supplement: Supplementary file 1 — Figure S1. Flow chart for the selection of the final sample Figure S2. Penalized cubic splines analyses for the association of HGS with (A) depression, and (B) anxiety incidence, additionally adjusted for physical activity. Data is presented as hazard ratio (thick blue line) and their 95% CI (shaded areas). Participants with depression, anxiety or mental health conditions at baseline were excluded from the analyses. A 2‐years landmark analysis was applied. Analyses were adjusted for age, sex, deprivation index, ethnicity, smoking status, alcohol intake, walking pace, TV viewing, sleep time, dietary intake (fruit and vegetables, red meat, processed meat and oily fish intake), body mass index, multimorbidity, and total physical activity (METs/min/week).). Table S1. Risk of depression and anxiety incidence according to HGS, additionally adjusted for total physical activity Figure S3. Penalized cubic splines analyses for the association of HGS with (A) depression, and (B) anxiety incidence, excluding from analyses participants with cardiovascular disease (CVD), cancer, and chronic obstructive pulmonary disease (COPD). Data is presented as hazard ratio (thick blue line) and their 95% CI (shaded areas). Participants with depression, anxiety or mental health conditions at baseline were excluded from the analyses. A 2‐years landmark analysis was applied. Analyses were adjusted for age, sex, deprivation index, ethnicity, smoking status, alcohol intake, walking pace, TV viewing, sleep time, dietary intake (fruit and vegetables, red meat, processed meat and oily fish intake), body mass index, and multimorbidity. Table S2. Risk of depression and anxiety incidence according to HGS, after excluding participants with cardiovascular disease (CVD), cancer, and chronic obstructive pulmonary disease (COPD) Table S3. Risk of depression and anxiety incidence according to muscle weakness and sarcopenia [file JCSM-13-1983-s001.docx]

**Supplementary Materials**

Supplementary Figure S1 p. 2

Supplementary Figure S2 p. 3

Supplementary Table 1 p. 4

Supplementary Figure S3 p. 5

Supplementary Table 2 p. 6

Supplementary Table 3 p. 7


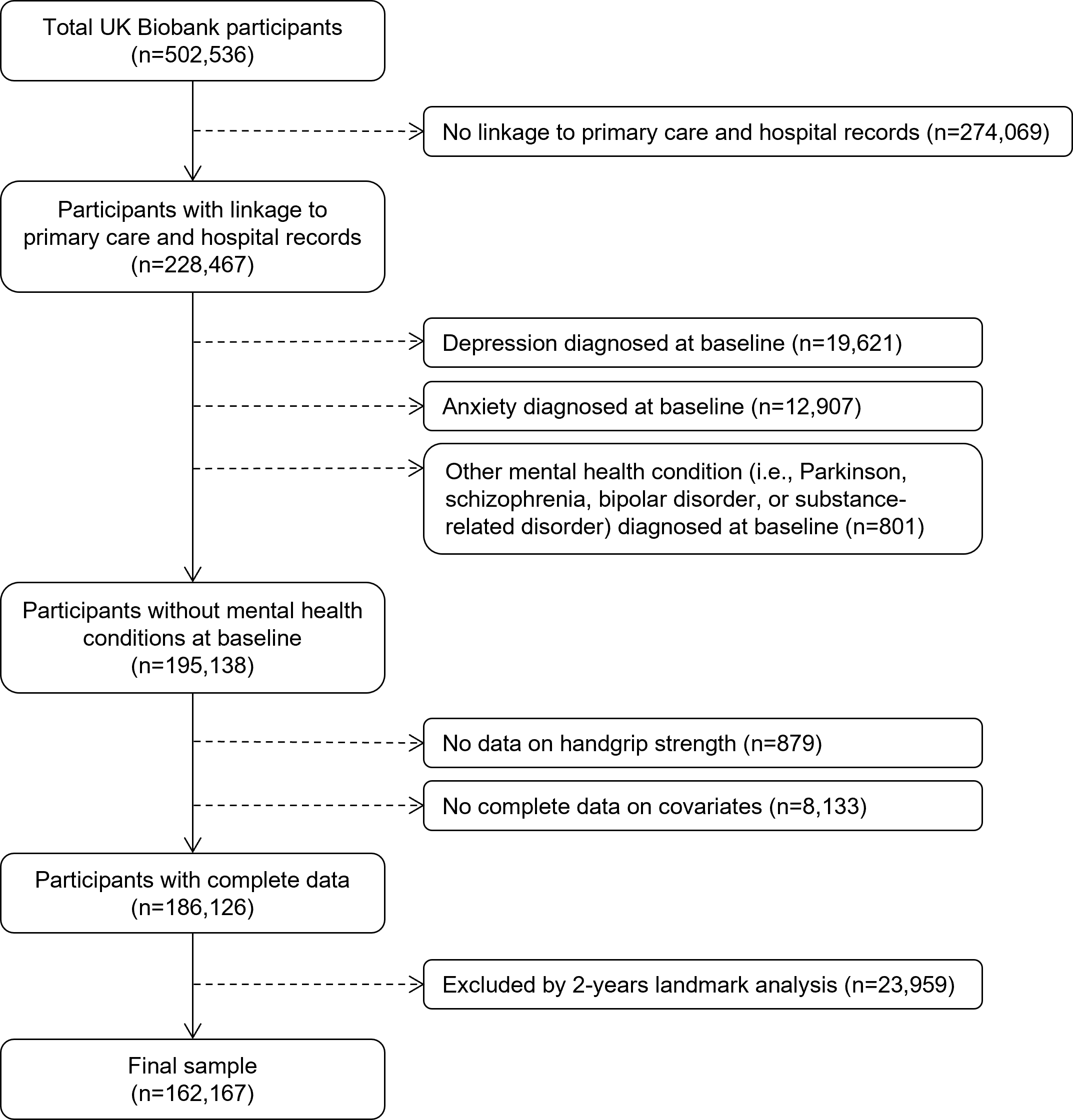


**Supplementary Figure S1.** Flow chart for the selection of the final sample

**
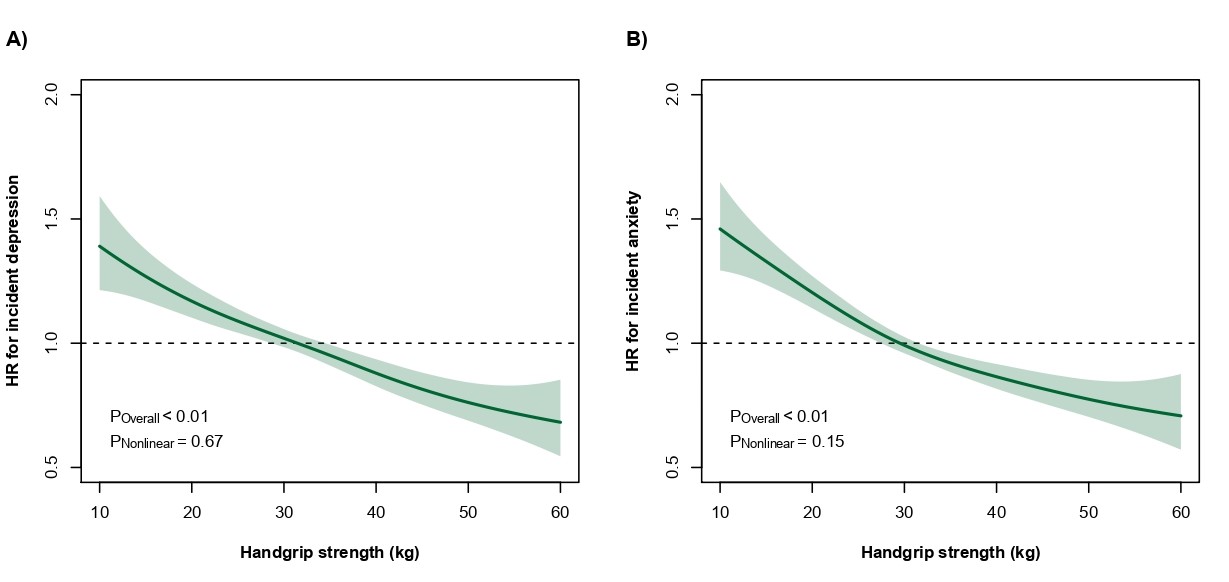
****Supplementary Figure S2.** Penalized cubic splines analyses for the association of HGS with (A) depression, and (B) anxiety incidence, additionally adjusted for physical activity.

Data is presented as hazard ratio (thick blue line) and their 95% CI (shaded areas). Participants with depression, anxiety or mental health conditions at baseline were excluded from the analyses. A 2-years landmark analysis was applied. Analyses were adjusted for age, sex, deprivation index, ethnicity, smoking status, alcohol intake, walking pace, TV viewing, sleep time, dietary intake (fruit and vegetables, red meat, processed meat and oily fish intake), body mass index, multimorbidity, and total physical activity (METs/min/week).

**Supplementary Table 1**. Risk of depression and anxiety incidence according to HGS, additionally adjusted for total physical activity

|  |  | **Depression incidence** | |  | **Anxiety incidence** | |
| --- | --- | --- | --- | --- | --- | --- |
| Grip strength |  | No. participants/ incidence | HR (95% CI) |  | No. participants/ incidence | HR (95% CI) |
| Tertile 3 (Highest) |  | 44,618/1,235 | Ref. |  | 44,618/1,514 | Ref. |
| Tertile 2 (Medium) |  | 44,034/1,397 | **1.13 (1.05, 1.22)** |  | 44,034/1,724 | **1.13 (1.05, 1.21)** |
| Tertile 1 (Lowest) |  | 46,574/1,699 | **1.22 (1.13, 1.31)** |  | 46,574/2,103 | **1.28 (1.19, 1.37)** |
| HR for trend |  | 135,226/4,331 | **1.10 (1.06, 1.14)** |  | 135,226/5,341 | **1.13 (1.09, 1.17)** |
| P for trend |  |  | **<0.001** |  |  | **<0.001** |
| Per 5-kg lower HGS |  | 135,226/4,331 | **1.07 (1.05, 1.10)** |  | 135,226/5,341 | **1.08 (1.06, 1.11)** |
| P value |  |  | **<0.001** |  |  | **<0.001** |

HR: Hazard ratio; CI: Confidence Interval. Analyses were adjusted for age, sex, deprivation index, ethnicity, smoking status, alcohol intake, walking pace, TV viewing, sleep time, dietary intake (fruit and vegetables, red meat, processed meat and oily fish intake), body mass index, multimorbidity, and total physical activity (METs/min/week). Significant values (p<0.05) ​​are bolded.

**
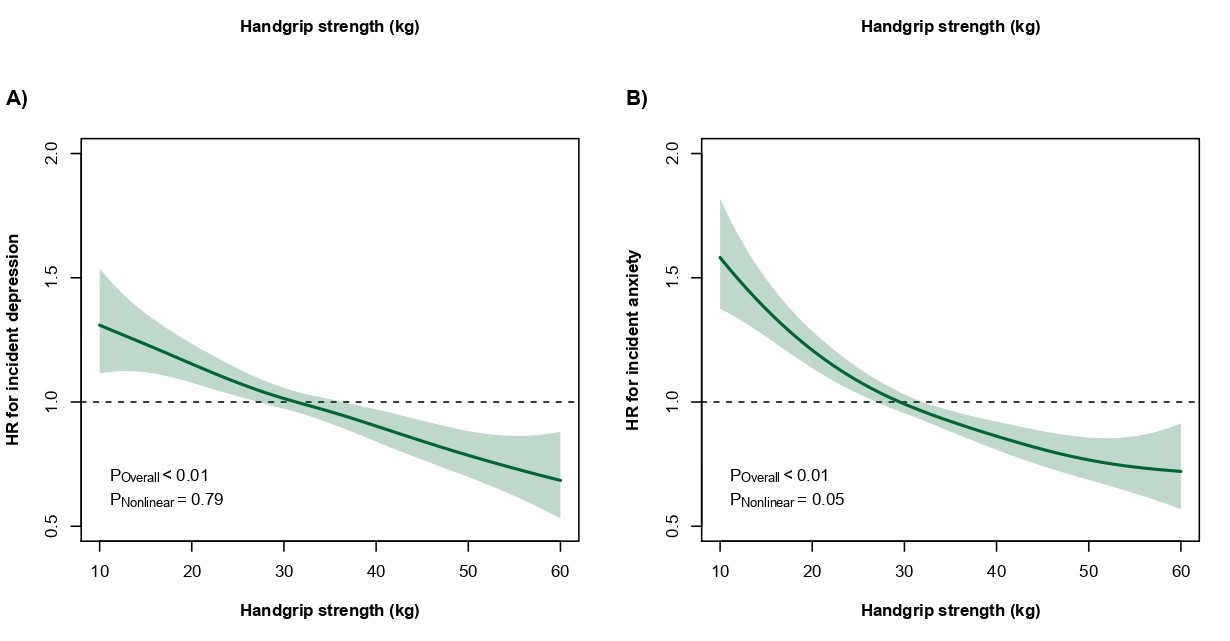
****Supplementary Figure S3.** Penalized cubic splines analyses for the association of HGS with (A) depression, and (B) anxiety incidence, excluding from analyses participants with cardiovascular disease (CVD), cancer, and chronic obstructive pulmonary disease (COPD).

Data is presented as hazard ratio (thick blue line) and their 95% CI (shaded areas). Participants with depression, anxiety or mental health conditions at baseline were excluded from the analyses. A 2-years landmark analysis was applied. Analyses were adjusted for age, sex, deprivation index, ethnicity, smoking status, alcohol intake, walking pace, TV viewing, sleep time, dietary intake (fruit and vegetables, red meat, processed meat and oily fish intake), body mass index, and multimorbidity.

**Supplementary Table 2**. Risk of depression and anxiety incidence according to HGS, after excluding participants with cardiovascular disease (CVD), cancer, and chronic obstructive pulmonary disease (COPD)

|  |  | **Depression incidence** | |  | **Anxiety incidence** | |
| --- | --- | --- | --- | --- | --- | --- |
| Grip strength |  | No. participants/ incidence | HR (95% CI) |  | No. participants/ incidence | HR (95% CI) |
| Tertile 3 (Highest) |  | 35,944/937 | Ref |  | 35,944/1,166 | Ref |
| Tertile 2 (Medium) |  | 35,516/1,074 | **1.15 (1.05, 1.25)** |  | 35,516/1,348 | **1.15 (1.06, 1.24)** |
| Tertile 1 (Lowest) |  | 36,097/1,251 | **1.25 (1.14, 1.36)** |  | 36,097/1,590 | **1.32 (1.22, 1.42)** |
| HR for trend |  | 107,557/3262 | **1.12 (1.07, 1.16)** |  | 107,557 (4,104) | **1.15 (1.10, 1.19)** |
| P for trend |  |  | **<0.001** |  |  | **<0.001** |
| Per 5-kg lower HGS |  | 107,557/3262 | **1.06 (1.03, 1.09)** |  | 107,557 (4,104) | **1.09 (1.07, 1.12)** |
| P-value |  |  | **<0.001** |  |  | **<0.001** |

HR: Hazard ratio; CI: Confidence Interval. Analyses were adjusted for age, sex, deprivation index, ethnicity, smoking status, alcohol intake, walking pace, TV viewing, sleep time, dietary intake (fruit and vegetables, red meat, processed meat and oily fish intake), body mass index, and multimorbidity. Significant values (p<0.05) are bolded.

**Supplementary Table 3**. Risk of depression and anxiety incidence according to muscle weakness and sarcopenia

|  |  | Cut-points by the FNIH Sarcopenia Project^a^ | | | | | | | | | | |
| --- | --- | --- | --- | --- | --- | --- | --- | --- | --- | --- | --- | --- |
|  |  | **Depression incidence** | | | |  | | **Anxiety incidence** | | | | |
|  |  | No. participants/ incidence | Model 1  HR (95% CI) | Model 2  HR (95% CI | Model 3  HR (95% CI | |  | | No. participants/ incidence | Model 1  HR (95% CI) | Model 2  HR (95% CI | Model 3  HR (95% CI |
| Non muscle weakness |  | 150,213/4,890 | Ref. | Ref. | Ref. | |  | | 150,213/5,912 | Ref. | Ref. | Ref. |
| Muscle weakness |  | 11,954/572 | **1.47**  **(1.35, 1.61)** | **1.25**  **(1.14, 1.37)** | **1.16**  **(1.06, 1.27)** | |  | | 11,954/702 | **1.43**  **(1.32, 1.55)** | **1.32**  **(1.22, 1.43)** | **1.24**  **(1.14, 1.35)** |
|  |  | Cut-points by the EWGSOP for muscle weakness^b^ | | | | | | | | | | |
| Non muscle weakness |  | 149,462/4,869 | Ref. | Ref. | Ref. | |  | | 149,462/5,889 | Ref. | Ref. | Ref. |
| Muscle weakness |  | 12,705/593 | **1.46**  **(1.34, 1.59)** | **1.24**  **(1.13, 1.36)** | **1.16**  **(1.06, 1.26)** | |  | | 12,705/725 | **1.42**  **(1.32, 1.54)** | **1.31**  **(1.21, 1.42)** | **1.23**  **(1.14, 1.34)** |

HR: Hazard ratio; CI: Confidence Interval. Model 1 was adjusted for age, sex, deprivation index, and ethnicity. Model 2 was adjusted for model 1 plus lifestyle factors, including smoking status, alcohol intake, walking pace, TV viewing, sleep time, and dietary intake (fruit and vegetables, red meat, processed meat and oily fish intake). Model 3 (fully adjusted) was adjusted as in model 2 plus body mass index, and multimorbidity. Significant values (p<0.05) ​​are bolded. ^a^Cut-points of grip strength <26 kg for men and <16 kg for women were used to defining clinically relevant muscle weakness, as recommended by the Foundation for the National Institutes of Health (FNIH) Sarcopenia Project [13, 20]; ^b^Cut-points of grip strength <27 kg for men and <16 kg for women were used to defining clinically relevant muscle weakness, as recommended by the European Working Group on Sarcopenia in Older People (EWGSOP) [21].
